# 893. Evaluating Weight Gain in Treatment-naïve, HIV-infected Patients Started on Antiretroviral Therapy

**DOI:** 10.1093/ofid/ofab466.1088

**Published:** 2021-12-04

**Authors:** Nimra Chaudhry, Eris Cani, Lendelle Raymond, Tae Park, Timothy Kanter

**Affiliations:** BronxCare Health System, Brooklyn, New York

## Abstract

**Background:**

There is increasing evidence that integrase strand transfer inhibitors (INSTIs) are associated with more weight gain when compared to other antiretroviral (ART) classes. Thus, the primary objective of the study was to evaluate the difference in weight gain at 6 and 18 months among treatment-naïve patients started on an INSTI-based versus a non-INSTI-based ART regimen.

**Methods:**

This was a retrospective cohort study of ART-naive adults who were initiated and maintained on INSTI and non-INSTI based regimens for at least 18 months at an HIV clinic in an inner-city hospital from January 2013 to June 2019. The non-INSTI-based regimens were darunavir (DRV) or rilpivirine (RPV)-based. Data collected included patient demographics, ART regimen, pre- and post-ART initiation weight in kilograms (kg), body mass index (BMI), CD4 count, and viral load. A two-tailed t-test was used to compare change in weight in INSTI-based versus non-INSTI-based regimens. Sub-group analyses were conducted using the ANOVA test.

**Results:**

Out of 170 patients, 60% were initiated on an INSTI-based regimen, 7.1% on a DRV-based regimen, and 32.9% on a RPV-based regimen. Of the patients initiated on INSTI-based regimens, 73.5% were on elvitegravir (EVG),16.7% on dolutegravir, 8.8% on bictegravir, and 0.98% on raltegravir. The mean age at ART initiation was 38 years with majority of the patients described as Black. More male patients received an INSTI-based regimen compared to females (77.5% vs. 32%). The average change in weight at 6 and 18 months in the INSTI-based group vs non-INSTI-based group was +3.6 kg vs. +2.9 kg (95% CI -2.2-0.7, p=0.317) and +5.7 kg vs.+4.8 kg (95% CI -3.2-1.2, p=0.357) respectively. There was no significant average change in weight among the INSTI-based regimens (+3.6 kg), vs DRV (+5.3 kg), or RPV (+2.4 kg) based regimens at 6 months (p=0.108) and 18 months [(+5.7 kg) vs (+7.2 kg), vs (+4.3 kg) (p=0.186) respectively]. Among the INSTIs, EVG was associated with the highest increase in weight at both 6 and 18 months (+3.9 kg and +5.8 kg). Forty-six percent of patients in the INSTI group were on tenofovir alafenamide (TAF) while no patients received TAF in the non-INSTI groups.

**Conclusion:**

When comparing INSTI-based to DRV or RPV-based regimens, there was no significant increase in average weight at 6 and 18 months.

**Disclosures:**

**All Authors**: No reported disclosures

